# Is integration and survival of newborn neurons the bottleneck for effective neural repair by endogenous neural precursor cells?

**DOI:** 10.3389/fnins.2014.00029

**Published:** 2014-02-20

**Authors:** Ann M. Turnley, Harleen S. Basrai, Kimberly J. Christie

**Affiliations:** Department of Anatomy and Neuroscience, The University of MelbourneParkville, VIC, Australia

**Keywords:** adult neurogenesis, neural stem cells, neural repair, SVZ, SGZ, hippocampus, olfactory bulb, neurite outgrowth

## Abstract

After two decades of research the existence of adult neural precursor cells and the phenomenon of adult neurogenesis is well established. However, there has been little or no effective harnessing of these endogenous cells to promote functional neuronal replacement following neural injury or disease. Neural precursor cells can respond to neural damage by proliferating, migrating to the site of injury, and differentiating into neuronal or glial lineages. However, after a month or so, very few or no newborn neurons can be detected, suggesting that even though neuroblasts are generated, they generally fail to survive as mature neurons and contribute to the local circuitry. Is this lack of survival and integration one of the major bottlenecks that inhibits effective neuronal replacement and subsequent repair of the nervous system following injury or disease? In this perspective article the possibility that this bottleneck can be targeted to enhance the integration and subsequent survival of newborn neurons will be explored and will suggest some possible mechanisms that may need to be modulated for this to occur.

## Introduction

Two decades of research has demonstrated that a surprisingly wide variety of factors can influence adult neural precursor cell biology (Christie and Turnley, 2012). This includes extrinsic factors, such as growth factors, cytokines, chemokines, neurotrophins, steroids and extracellular matrix molecules as well as cell intrinsic factors such as transcription factors and signal transduction pathway regulators (Christie and Turnley, 2012; Christie et al., 2013a). In general, endogenous adult neural precursor cells can be quite easily induced to proliferate and migrate, and depending on the context, differentiate into neuronal or glial cell types. However, fewer factors have been identified that induce newborn neurons to integrate into the local circuitry and survive more than a few weeks after their birth. Indeed at least 50% of newborn neurons fail to survive longer than a month or two after their generation (Petreanu and Alvarez-Buylla, 2002; Dayer et al., 2003). This makes sense under normal physiological conditions, where newborn neurons replenish local neurons lost due to normal turnover, to homeostatically maintain neuron numbers (Valley et al., 2009). Addition of newborn neurons to existing circuitry has specific functional outcomes. In the olfactory bulb, addition of new neurons is required for short-term olfactory memory, perceptual learning, and for innate olfactory responses (Breton-Provencher et al., 2009; Moreno et al., 2009; Sakamoto et al., 2011). In the hippocampus, adult neurogenesis plays roles in anxiety and affective behaviors, cognition and spatial memory (Ming and Song, 2011), and is proposed to be vital for forgetting of hippocampal-dependent short-term memories (Frankland et al., 2013). However, in instances of larger neuronal loss, such as following injury or disease, this failure of newborn neurons to increase their integration and survival in conjunction with increases in proliferation and redirected migration means that the full potential of adult neural progenitor cells (NPCs) to repair the damage may not be realized. This perspective article will explore some of the mechanisms and factors that may be targeted to enhance newborn neuron survival, summarized in Table 1.

**Table 1:** **Factors that regulate or may be targeted to promote survival of adult newborn neurons**.

**Factor**	**Role**	**References**
**EXOGENOUS**		
Neurotransmitters—GABA and glutamate	Activity induced survival and synaptic integration	Gascon et al., 2006; Ge et al., 2006; Platel et al., 2010; Kim et al., 2012; Chancey et al., 2013
Neurotrophins—BDNF	Enhances neurite outgrowth, dendritic arborization, and spine density	Miyamoto et al., 2006; Cheung et al., 2007; Chan et al., 2008; Gao and Chen, 2009; Gao et al., 2009; Bergami et al., 2013
**ENDOGENOUS**		
Rho GTPases	Cytoskeletal reorganization—dendrite/axon outgrowth, dendritic spine formation—regulation of plasticity induced survival	Nikolic, 2002; Keung et al., 2011; Christie et al., 2013b; Vadodaria et al., 2013
	NPC migration	Leong et al., 2011
	Extant neuron survival in stroke and Parkinson's disease models	Lemmens et al., 2013; Rodriguez-Perez et al., 2013
SOCS2	Regulation of growth factor signaling and neurite outgrowth	Goldshmit et al., 2004a,b; Basrai et al., 2013
**TRANSCRIPTION FACTORS**		
zif268/egr1, KLF9, NeuroD1, cAMP response element, ATF5, miR-132	Regulation of neuronal morphology and maturation	Giachino et al., 2005; Gao et al., 2009; Jagasia et al., 2009; Scobie et al., 2009; Pathania et al., 2012; Wang et al., 2012; Veyrac et al., 2013
p63	Anti-apoptotic	Cancino et al., 2013
NFATc4	Mediates BDNF-induced survival	Quadrato et al., 2012
**OTHER POTENTIAL MODULATORS**		
Ephs/ephrins	Regulation of axonal and dendritic sprouting, synaptic plasticity	Goldshmit et al., 2011; Overman et al., 2012; Spanevello et al., 2013
Peri-neuronal nets	Inhibits synaptic plasticity; degradation promotes plasticity	Kwok et al., 2011; Wang et al., 2011
Environmental enrichment/ Forced use	Enhances synaptic plasticity	Rochefort et al., 2002; Miwa and Storm, 2005; Yamaguchi and Mori, 2005; Alonso et al., 2006; Mandairon et al., 2006; Overman et al., 2012

## Source of neural stem/progenitor cells—subventricular zone vs. hippocampus

There are two principle sources of neural progenitor cells in the adult brain: the subventricular zone (SVZ) lining the lateral walls of the lateral ventricles, which supplies new neurons to the olfactory bulb and the subgranular zone (SGZ) of the hippocampal dentate gyrus, which produces new hippocampal granule cell neurons. Under normal physiological conditions, both of these NPC populations primarily generate neurons, however there are differences in the neuroblasts and neurons they produce and their response to neural damage. SVZ-derived cells must migrate a long distance along the rostral migratory stream to reach the olfactory bulb, while hippocampal-derived cells only migrate a short distance from the SGZ into the granule cell layer directly adjacent. In response to neural damage, both populations increase their proliferation but apparently only SVZ-derived cells migrate to ectopic sites of damage (Ming and Song, 2011), while hippocampal cells remain within the granule cell layer. The differentiation fate of SVZ-derived cells is also more easily switched to a glial fate in response to damage and the subsequent production of chemokines and cytokines, while SGZ cell fate remains largely neuronal depending on type of damage (Christie and Turnley, 2012). However the end stage of neuronal integration and survival does not appear to differ greatly, with the majority of neurons failing to survive in both cases.

## Adult newborn neuronal survival under normal physiological conditions

### Integration of newborn neurons into existing local circuitry

While there are homeostatic mechanisms in place to regulate the integration and subsequent survival of newborn neurons into local circuits under normal physiological conditions, this does not mean to imply that this process is always held at a constant level. Indeed, the numbers of newborn neurons that survive and integrate can be varied by altered levels of circuit activity/plasticity. In the olfactory bulb, alteration of local activity by complex odor environments or odor discrimination learning increases the survival of newborn neurons (Rochefort et al., 2002; Miwa and Storm, 2005; Alonso et al., 2006; Mandairon et al., 2006), while conversely at the critical time of neuronal integration, sensory deprivation and hence decreased circuit activity results in increased newborn neuron death (Yamaguchi and Mori, 2005). Similarly, numerous activity and environment-induced alterations in adult hippocampal neurogenesis have been described, including exercise, environmental enrichment and learning increasing newborn neuron numbers, with stress, steroids, and depression decreasing them. However, with some exceptions, these factors alter final mature newborn numbers by increasing or decreasing proliferation of neural precursor cells and hence production of new neurons, rather than acting primarily at the integration and survival stage. It seems that the newborn hippocampal neurons, during the critical few weeks after their generation, require activity in the form of effortful learning, to promote their survival (Shors et al., 2012).

So, how do newborn neurons integrate into the extant local circuitry? While the mechanisms are not yet well understood it seems that newly generated neurons compete for synaptic space, with only those that make successful connections surviving. The newborn neurons must compete not only with each other but also with older neurons to integrate into the circuitry. It should be noted that the regions into which newborn neurons integrate are actually open to the competition. In other words, they are very plastic regions of the CNS that unlike other CNS regions, are primed to allow new neurons to be added to the circuitry. This is highlighted in neural precursor cell transplantation studies, whereby new neurons were produced and survived if transplanted into neurogenic regions but not into non-neurogenic regions (Suhonen et al., 1996; Shihabuddin et al., 2000). While there are similarities in regulation of neuronal survival and integration of SVZ- and SGZ-derived neurons under normal physiological conditions, there are quite important differences following injury. This includes their ability to migrate to distant sites of damage and differences into which they integrate. The SVZ-derived cells will migrate to sites of damage in non-neurogenic regions, e.g., cortex or striatum and therefore need to try to integrate and survive in areas that are usually non-receptive to such processes. SGZ-derived cells remain in a neurogenic environment, although injury-induced plasticity may be increased (Perederiy et al., 2013). In the injured non-neurogenic CNS there is a variable degree of rewiring and plasticity occurring, which may change the normally non-neurogenic environment to a partially neurogenic environment in some instances and which may account for the various reports of newborn neurons at sites of injury or disease (Christie and Turnley, 2012). So, what can be taken from our knowledge of factors that regulate normal physiological adult newborn neuron integration and survival and be applied to the injured regions of the CNS?

### Factors that enhance newborn neuron integration and survival under normal physiological conditions

Compared to our knowledge of factors that promote adult neural precursor cell proliferation or differentiation, there is comparatively little known about factors that promote newborn neuron survival and integration *per se*. Broadly speaking, what information we do have centers on ways to alter plasticity—particularly at the synaptic or cell morphological level (dendrite and axon morphology). These may be categorized into factors that regulate circuit/neuronal activity (e.g., neurotransmitters) and factors that regulate cellular morphology (e.g., cytoskeletal regulators, neurotrophins), as well as less-defined but otherwise acknowledged general regulators of neural plasticity, such as environmental enrichment and use/activity induced plasticity.

These different signals converge on a range of transcription factors, the roles, and regulation of which are beginning to be elucidated (Christie et al., 2013a). These include transcription factors that regulate neurochemical and/or morphological maturation, such the inducible immediate early gene zif268/egr1 (Veyrac et al., 2013), KLF9 (Scobie et al., 2009), NeuroD1 (Gao et al., 2009), the cAMP response element (Giachino et al., 2005; Jagasia et al., 2009) and ATF5 (Wang et al., 2012), as well as the microRNA miR-132 (Pathania et al., 2012). The anti-apoptotic p63 (Cancino et al., 2013) and NFATc4, which mediates BDNF-dependent survival (Quadrato et al., 2012), also promote newborn neuron survival.

#### Neurotransmitters

Early in the life of a newborn neuron, before integration has taken place, the cells are responsive to non-synapse-mediated GABA stimulation. After further maturation, the cells become synoptically responsive to GABA with concomitant responsiveness to glutamate (Gascon et al., 2006; Ge et al., 2006; Kim et al., 2012; Chancey et al., 2013). It has been proposed that this responsiveness may allow synaptic integration into local circuitry to occur in a process whereby a highly motile filopodia of a newborn neuron contacts a pre-existing synapse, possibly in response to excess neurotransmitter release. This connection then matures, becomes stabilized and the filopodium develops into a mature dendritic spine. Astrocyte-released glutamatergic activation of neuroblast NMDA receptors has been shown to be required for synaptic integration (Platel et al., 2010), while the level of cell-intrinsic excitability also modulates survival (Lin et al., 2010). For such an interaction to be most effective, there likely needs to be a substantial degree of synaptic and dendritic remodeling occurring, as in the plastic neurogenic regions of the CNS, which requires substantial cytoskeletal rearrangement and regulation. Enhancing plasticity by enhancing cytoskeletal rearrangement provides another potential target for increasing the likelihood of integration and survival of newborn neurons.

#### Cytoskeletal rearrangement as a potential target for enhancing newborn neuron integration into local circuitry—modulating the RhoA family of small GTPases

While a multitude of factors have effects on cytoskeletal rearrangement, most of these converge on the family of small Rho GTPases, RhoA, Rac1, and cdc42. The Rho GTPases are regulators of neurite outgrowth, with RhoA activation inhibiting and Rac1 activation promoting neurite outgrowth and dendritic spine formation (Nikolic, 2002). Enhancement of a newborn neuron's ability to produce dendrites, axons, or dendritic spines may give it a competitive advantage, while increasing synaptic remodeling or turnover of existing local circuitry could also increase the chances of newborn neuron integration. A number of recent studies implicate these molecules in regulation of adult newborn hippocampal neuron maturation and survival, as recently reviewed (Vadodaria and Jessberger, 2013).

Conceptually, Rac1 may promote neuronal survival and integration while RhoA may oppose it, given the promotion and inhibition of neurite outgrowth and spine formation respectively. This indeed appears to be the case. Deletion or inhibition of Rac1 or cdc42 blocked dendrite and spine formation of adult hippocampal newborn neurons, although there were no effects on neuron survival (Vadodaria et al., 2013). Conversely, constitutive activation of RhoA decreased the percentage of newborn neurons (Keung et al., 2011), while inhibition of RhoA signaling specifically enhanced the survival of newborn hippocampal neurons, which correlated with enhanced spatial memory (Christie et al., 2013b). Rho kinase inhibition also promotes ectopic migration of SVZ-derived neural precursor cells and subsequent neuron survival (Leong et al., 2011). Therefore, activation of Rac1 or cdc42 or inhibition of RhoA signaling pathways may be of potential therapeutic effectiveness at promoting survival and integration of newborn neurons. Results are yet to be reported on effects on neurogenesis, however use of the Rho kinase inhibitor HA1077 (Fasudil) promotes extant neuron survival, regeneration and functional improvement, in Parkinson's disease and stroke models (Lemmens et al., 2013; Rodriguez-Perez et al., 2013).

### Factors that regulate neuronal morphology and survival—other potential targets to enhance survival and integration of newborn adult neurons

While administration of Fasudil appeared to have few side effects, not many studies have been performed at present and so the possibility exists that problems may emerge. A number of other factors are known to regulate neuronal morphology and also play a role in neurogenesis and newborn neuron survival. These may, at least in part, signal via Rho GTPases to perform their function, but given the broad expression of Rho GTPases, in some instances it may be more prudent to target molecules that have a more limited expression profile, to avoid potential side effects. These have been recently reviewed (Vadodaria and Jessberger, 2013) so only a subset will be discussed briefly here.

#### SOCS2

An example of such a molecule is the intracellular regulator of cytokine and growth factor signaling, Suppressor of Cytokine Signaling-2 (SOCS2). Although best known as a negative feedback regulator of growth hormone signaling (Metcalf et al., 2000), it has a broader range of molecules it interacts with, including EGF and Epo (Basrai et al., 2013; Goldshmit et al., 2004a,b). Overexpression of SOCS2 enhances neurite length and branching, which may in part be due to activation of Rac1 and inhibition of Rho kinase (Goldshmit et al., 2004a,b). During development, SOCS2 is expressed widely throughout the developing brain and regulates developmental neurogenesis (Polizzotto et al., 2000; Turnley et al., 2002). In the adult its expression is primarily in the dentate gyrus and hippocampal CA3 region, suggesting it plays a role in adult neurogenesis. Overexpression of SOCS2 increased numbers of newborn adult hippocampal neurons without affecting their differentiation or proliferation of neural precursor cells (Ransome and Turnley, 2008). These results are in keeping with the idea that enhancing neurite outgrowth and complexity may enhance survival and integration of newborn neurons.

#### Neurotrophins

Although many growth factors have been shown to play a role in some aspect of adult neurogenesis, often promoting proliferation and survival of neural precursor cells (Christie and Turnley, 2012), few have been shown to play a role in the specific stage of neuronal maturation, integration and survival. The neurotrophin brain-derived neurotrophic factor, BDNF, is one such factor. BDNF has been shown to regulate neuronal morphology via activation of the Rho GTPases Rac1 and cdc42 (Miyamoto et al., 2006; Cheung et al., 2007). It is important for neurite outgrowth, dendritic arborization and spine density of adult hippocampal neurons, but not proliferation or cell fate specification (Chan et al., 2008; Gao and Chen, 2009; Gao et al., 2009; Bergami et al., 2013) and deletion of TrkB decreases survival and induces an anxiety-like phenotype (Bergami et al., 2008). Further, in the absence of hippocampal BDNF, there is increased death of adult newborn hippocampal neurons following traumatic brain injury (Gao and Chen, 2009).

## Other modulators of neural plasticity that may be harnessed to promote survival and integration

Normal physiological levels of neurogenesis, newborn neuron integration, and survival are enhanced by activity-induced plasticity. This can be applied to the therapeutic scenario, where environmental enrichment, exercise, or forced use can induce circuit sprouting and plasticity and lead to functional improvement. While this has been examined more in terms of axonal regeneration, such as by inhibition of repulsive axon guidance molecules, it is also applicable to enhancement of neuron integration into damaged circuitry. For example, blocking of members of the Eph/ephrin family promotes axonal and dendritic sprouting/regeneration and enhances functional recovery (Goldshmit et al., 2011; Overman et al., 2012; Spanevello et al., 2013). This sprouting/plasticity can be further enhanced with forced use of the affected limb (i.e., enhancing plasticity) in a stroke model (Overman et al., 2012). Altering the expression of different Ephs or ephrins has positive or negative effects on neurogenesis (Ricard et al., 2006; Chumley et al., 2007; Jiao et al., 2008; Hara et al., 2010; Murai et al., 2010; Ashton et al., 2012). It is likely that their neuronal progeny also express these molecules, suggesting that blocking them or modulating their signaling pathways (again, converging on Rho GTPases) may enhance newborn neuron survival and integration into damaged but sprouting local circuitry. Indeed, deletion of ephrinB3 leads to enhanced neurogenesis around the lesion site in a stroke model, however it also resulted in more severe ischemic damage (Doeppner et al., 2011). Targeting of other repulsive guidance molecules, such as Semaphorins, Slits, and DCC may also be advantageous (Christie and Turnley, 2012).

Another factor that inhibits neural regeneration and limits access to neuronal circuits is the peri-neuronal net (Kwok et al., 2011). This is made up of extracellular matrix, such as proteoglycans, surrounding neuronal cell bodies, and their processes. Degradation of the nets, for example using chondroitinases, enhances sprouting, plasticity, and functional improvement after injury (Wang et al., 2011). Given that breakdown of the peri-neuronal nets makes it easier for synaptic plasticity to occur, it may also allow easier access and integration of newborn neurons, although this has yet to be examined.

## Combination of newborn neuron survival promoting agents with neuroprotective, proliferative, or plasticity inducing agents

While a number of factors have been described above that have the potential to enhance newborn neuron integration and survival in an injury or disease setting, we cannot forget that the damaged CNS environment is much different to the normal physiological neurogenic niches where neurogenesis normally takes place. On the plus side, the injured CNS undergoes a certain amount of sprouting and local circuit rearrangement, which may open up the opportunity for newborn neurons to compete for synaptic space along with sprouting extant neurons, unlike the comparatively non-plastic normal CNS parenchyma. On the minus side, the damaged CNS environment is quite an inhibitory environment, such that newborn neuroblasts may need to be protected so that they can differentiate and become mature neurons, rather than dying of other causes along the way. Neuroprotective or anti-inflammatory strategies probably also need to be included with therapy to improve newborn neuron maturation, integration, or plasticity. There are a number of promising molecules in this regard, such as erythropoietin (Epo) which is neuroprotective and enhances the numbers of neural precursor cells and neurons following neural injury (Shingo et al., 2001; Wang et al., 2004; Lu et al., 2005; Tsai et al., 2006; Byts and Siren, 2009; Zhang et al., 2012). It has shown promise in a large range of pre-clinical studies and is currently undergoing clinical trial for a range of conditions. Combinatorial studies now need to be performed, with for example Epo and inhibition of Rho kinase, to determine whether the outcome is further improved, and numbers of surviving and integrated newborn neurons is enhanced more than either factor alone.

## Conclusions

There is a high rate of failure of newborn adult neurons to integrate into local circuitry and survive under physiological conditions and an even higher failure rate in injury or disease conditions. We propose that provision of factors that enhance the competitiveness of newborn neurons to integrate into circuitry, either by affecting the newborn neurons themselves or by affecting plasticity of the local circuitry, will improve this bottleneck of low newborn neuron survival, leading to improved functional outcome following neural damage. Further, it is likely that such treatments will be more effective if combined with treatments that will make the injured CNS a less toxic environment, by use of inflammatory modulators or neuroprotective agents. After two decades of research we know what needs to be done and we now have several avenues of approach to be tested that may actually allow the therapeutic promise of adult neural stem cells to be realized.

### Conflict of interest statement

The authors declare that the research was conducted in the absence of any commercial or financial relationships that could be construed as a potential conflict of interest.
